# Surgical Resection of Brain Metastases—Impact on Neurological Outcome

**DOI:** 10.3390/ijms14058708

**Published:** 2013-04-24

**Authors:** Petra Schödel, Karl-Michael Schebesch, Alexander Brawanski, Martin Andreas Proescholdt

**Affiliations:** Department of Neurosurgery, University of Regensburg Medical Center, Franz Josef Strauß Allee 11, Regensburg 93053, Germany; E-Mails: petra.schoedel@klinik.uni-regensburg.de (P.S.); karl-michael.schebesch@klinik.uni-regensburg.de (K.-M.S.); alexander.brawanski@klinik.uni-regensburg.de (A.B.)

**Keywords:** resection, recurrence, survival, neurological status, prognosis, brain metastases

## Abstract

Brain metastases (BM) develop in about 30% of all cancer patients. Surgery plays an important role in confirming neuropathological diagnosis, relieving mass effects and improving the neurological status. To select patients with the highest benefit from surgical resection, prognostic indices (RPA, GPA) have been formulated which are solely focused on survival without considering neurological improvement. In this study we analyzed the impact of surgical resection on the neurological status in addition to overall survival in 206 BM patients. Surgical mortality and morbidity was 0.0% and 10.3% respectively. New neurologic deficits occurred in 6.3% of all patients. The median overall survival was 6.3 months. Poor RPA class and short time interval between diagnosis of cancer and the occurrence of BM were independent factors predictive for poor survival. Improvement of neurological performance was achieved in 56.8% of all patients, with the highest improvement rate seen in patients presenting with increased intracranial pressure and hemiparesis. Notably, the neurological benefits were independent from RPA class. In conclusion, surgical resection leads to significant neurological improvement despite poor RPA class and short overall survival. Considering the low mortality and morbidity rates, resection should be considered as a valid option to increase neurological function and quality of life for patients with BM.

## 1. Introduction

Brain metastases (BM) are by far the most frequent intracranial tumor in adults, outnumbering primary brain tumors by about four times [1]. Recently, an apparent increase in the incidence of brain metastases has been observed [2], which may be related to the higher sensitivity of modern neuroradiological imaging technology [3]. In specific cancer types, for which molecular treatment strategies are now available, a significantly higher percentage of BM have been detected [4]. Since BM generally occur late in the course of the disease, the prolonged overall survival induced by advanced treatment options allows for BM [5]. Alternatively, certain large-molecule biological agents such as trastuzumab do not properly penetrate the blood-brain barrier [6]. This leads to an organ specific failure of these molecular strategies due to micrometastases in the brain, which are sequestered behind the vascular barrier. In addition to poor overall survival, with a median survival time between 5 to 12 months [7], about 90% of all BM patients show neurological impairment which negatively impacts quality of life [8]. Surgical resection plays an important role in relieving mass effects and decompressing eloquent areas of the brain causing improvement of neurological status [9]. Recently, two randomized clinical trials demonstrated that surgical resection prolongs overall survival compared to whole brain radiation treatment (WBRT) alone [10,11]. To benefit from surgical resection, a patient must be medically suitable, with a disease prognosis amenable to benefit from local central nervous system tumor control [12]. This has led to the formulation of prognostic indices such as the RPA classification, categorizing patients with a Karnofsky performance score (KPS) of more than 70, age under 65 years, with controlled primary tumor and no extracranial metastases as most suitable for surgical resection [13,14]. However, these algorithms to select suitable patients for resection were solely focused on overall survival, without considering neurological improvement symptom relief and personal independence as an important endpoint for QOL, especially in a palliative setting [15]. Data regarding detailed evaluations of specific neurological impairment patterns and their response to surgical treatment were lacking. We therefore investigated the impact of surgical resection in a cohort of 206 BM patients on neurological status in addition to overall survival. We have also identified independent factors predictive for poor outcome in this patient population.

## 2. Materials and Methods

The study was approved by the University Regensburg Ethics Committee and conducted in accordance to the ethical standards of the Helsinki Declaration. Patient’s written consent was obtained whenever possible, and all study results were stored and analyzed anonymously. We analyzed a cohort of 206 patients (female/male: 84/122) who were initially diagnosed with metastatic brain disease at a mean age of 61.1 years (range: 23.4–83.9) and consecutively treated with microsurgical resection for BM. A detailed description of patient characteristics is provided in Table 1. Exclusion criteria were biopsy only, histology other than metastatic tumor and age younger than 18 years. Indication for surgical resection was based on either decompressing a mass lesion or establishing a histological diagnosis. Intraoperative ultrasound was used in 96.1% of all cases; neuronavigation and awake craniotomy with direct cortical and subcortical stimulation were used in 63.6% and 5.3%, respectively. Preoperative steroid medication was given in 95.6% of all cases, which was tapered postsurgically in 75.6% of all patients on steroid medication. All patients received MRI scanning prior to surgery and within 72 hours after surgery to evaluate the extent of resection (EOR), categorized as gross total resection (GTR, 92.2%) or subtotal resection (STR, 7.8%). In case of multiple metastases, the lesion with the largest mass effect leading to clinical impairment was resected. No more than 3 tumors were removed in one session. Intratumoral hemorrhage was detected in 16.5% on the preoperative MRI, leading to emergency evacuation of the tumor in 4.4% of all patients. Radiotherapy was administered as WBRT with 30–35 Gy to 64.6% of all patients, from which 18 patients (13.5%) received additional stereotactic boost radiation of the tumor bed. Adjuvant chemotherapy was administered postsurgically in 35.4% of all patients. From all patients, 9.7% corresponded to RPA class I, 77.7% and 12.6% to class II and III respectively. Follow up was completed up to March 2012 by reviewing outpatient records and contacting the patient, a family member or the patient’s primary physician. No patient was lost for follow up. The median follow up time was 6.1 months. Overall survival was analyzed using the Kaplan-Meier procedure, with Log-rank analysis utilized to calculate differences in overall survival. To isolate independent predictive factors for survival, multivariate analysis was performed using the Cox hazard regression model [16]. Quality of life and neurological deficits were quantified with the Medical Research Council Neurological Performance Score [17] (MRC-NPS; Table 2) and the Karnofsky Performance Score (KPS) respectively. Differences in improvement rates were analyzed by performing rates and proportions testing (chi square analyses). Additionally, signs of increased intracranial pressure (ICP), hemiparesis, visual deficits and aphasia were recorded preoperatively, at discharge and at the last follow up exam.

## 3. Results

**Figure 1:**
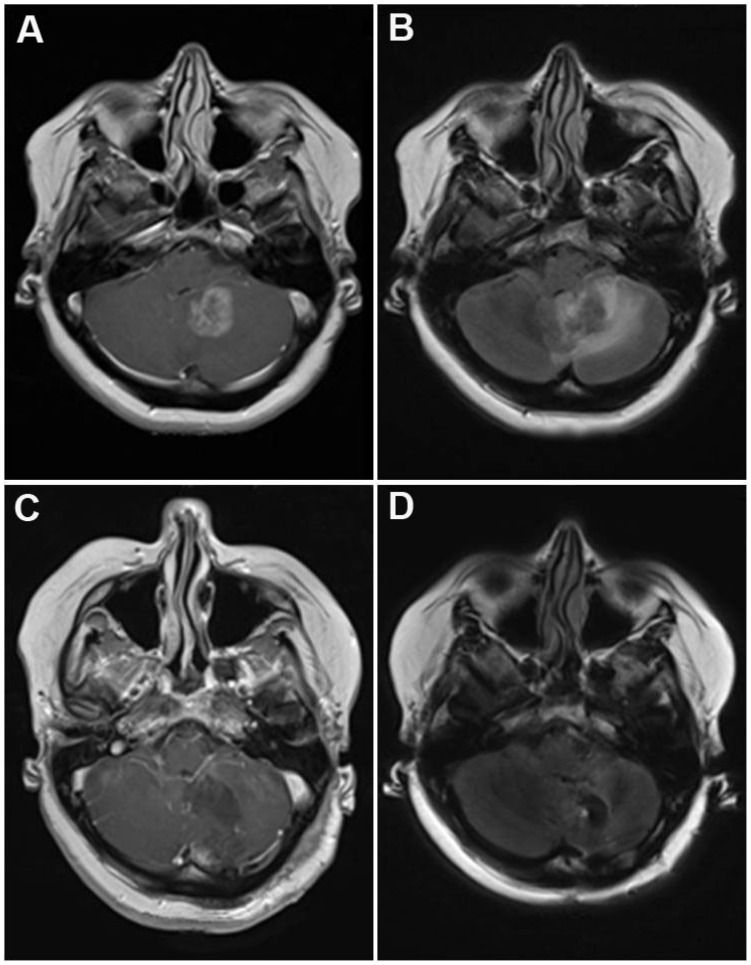
Preoperative MRI of a patient with a cerebellar metastasis from lung cancer utilizing (**A**) T1 weighted, contrast enhanced and; (**B**) fluid attenuated inversion recovery sequences (FLAIR). Note the mass effect on the fourth ventricle and the significant perifocal edema. Panel **C** & **D** displays the postoperative scan demonstrating the decompression of the CSF pathways and the reduced edema immediately after resection.

**Figure 2:**
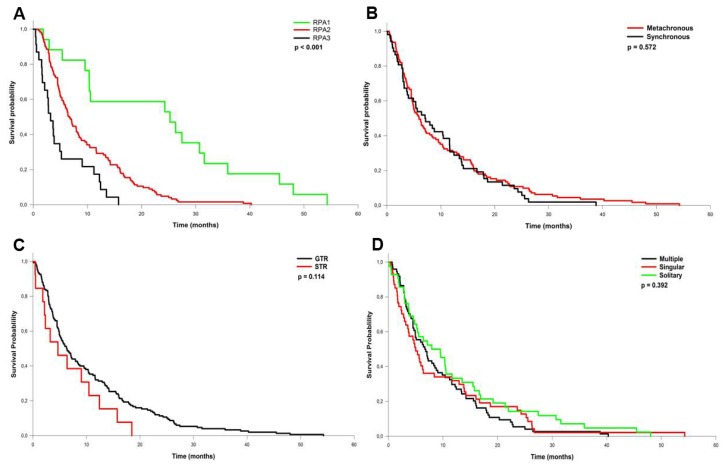
Kaplan–Meier curves of the overall survival in patients with brain metastases receiving surgical resection. (**A**) RPA classification is significantly related to survival (*p* < 0.001), whereas (**B**) synchronous or metachronous occurrence of metastases, (**C**) extent of resection (GTR = gross total resection, STR = subtotal resection), as well (**D**) the metastatic status (solitary, singular or multiple) is not (*p* > 0.05).

**Figure 3:**
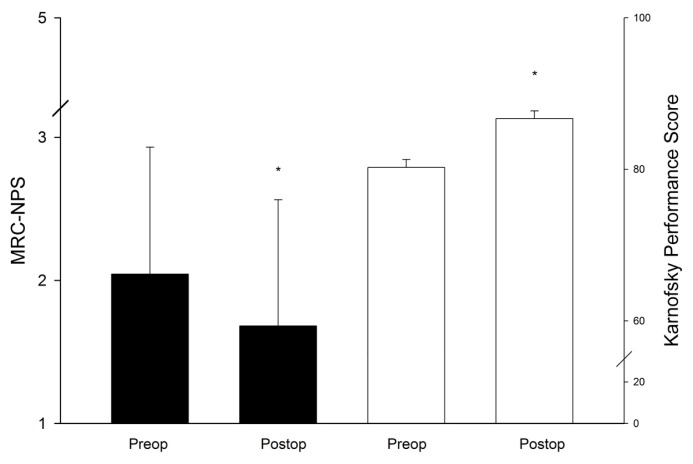
The bar graphs illustrate significant improvement of neurological status measured by the MRC-NPS system (black) and Karnofsky Performance Score (KPS) (white) after microsurgical resection of brain metastases (*p < 0.001).

The most frequent primary cancer types encountered were lung cancer (34%), malignant melanoma (14.5%), breast (13.6%) and colon cancer (9.7%) (Table 1). The most frequent location was the infratentorial compartment (Figure 1), followed by the frontal and parietal lobes. The median diameter of the tumors was 3.2 cm (range: 7.0–2.2 cm). Solitary metastases, defined as a single metastatic lesion in the brain without evidence for extracerebral metastases, occurred in 29.7% of all cases, 29.1% of all patients presented with a single metastatic lesion (*i.e.*, one brain metastasis with additional extracerebral metastases), while 41.2% of all patients had multiple brain metastases. Surgical mortality and morbidity was 0.0% and 10.3% respectively. New neurologic impairment or worsening of pre-existing deficits occurred in 6.3% of all patients resulting in an overall morbidity rate of 16.6% (Table 3). The local recurrence rate was 22.1% with a one-year recurrence rate of 18.5%. Post-surgical radiation therapy significantly reduced the recurrence rate (HR 2.2; 95% CI 1.2–4.3; *p* = 0.025), whereas the extent of resection had no influence on the risk of recurrence (HR 0.75; 95% CI 0.3–1.8; *p* = 0.114). The median overall survival was 6.3 months. Patients corresponding to RPA class I showed a significantly better median overall survival (25.2 months) compared to patients in class II (6.7 months) and III (3.2 months) (0 < 0.001, Table 4, Figure 2A). In contrast, no significant difference in overall survival was detected between metachronous *vs.* synchronous occurrence of BM, EOR, or solitary, single or multiple metastases (Figure 2B–D). Multivariate analysis revealed poor RPA class and a short time interval between initial diagnosis of cancer and the first occurrence of BM as independent factors predicting short survival (Table 5). The majority of all patients presented with an impaired KPS score (92.7%) which improved in 54.9% of all affected patients after surgery and at last follow up (*p* < 0.001; Figure 3). Similarly, the MCR-NPS rating was reduced indicating moderate to severe neurological deficits in 70.9% of the patients, which was again significantly improved in 56.8% (*p* < 0.001; Figure 3). The most frequent clinical symptoms in the study population were signs of increased ICP in 40.8% of all patients, which was completely resolved in 97.6% of all affected patients. Interestingly, hemiparesis, which occurred in 10.2% of the patients, was significantly improved postoperatively (*p* < 0.014); however visual field deficits and signs of aphasia were not improved by surgical resection (Table 6). The distribution of neurological impairment was significantly different within the RPA classes, with patients in class III presenting significantly more frequently with impaired MRC-NPS rating, hemiparesis and raised ICP. In contrast, the functional improvement rate was equally distributed throughout the RPA classes, indicating a significant benefit of neurological function and quality of life even in patients belonging to the worst prognostic group.

## 4. Discussion

Our study demonstrates that BM patients with a poor RPA rating resulting in a shorter overall survival time still show significant neuro-functional benefit from surgical resection. However, this analysis has several limitations: First, this is a retrospective design lacking the proper non - surgical control group. Second, both KPS and the MRC-NPS may not adequately reflect the exact neurocognitive status in this patient population. Third, volumetric data of tumor size is lacking which is important if comparing our surgical results against a radiosurgical series. These aspects need to be addressed in a future investigation using a prospective study design. According to the American Cancer Society, the 5-year survival rate for all cancers increased from 50% in 1974–1976 to 70% between 2000 and 2008 [18]. Advances in longer-term survival have even been greater for specific histologies such as breast cancer. However, the occurrence of brain metastases still marks the final stage of the disease accompanied with an exceptionally poor prognosis [7]. Two randomized clinical trials have demonstrated that surgical resection is superior to WBRT only [10,11], and that WBRT after resection significantly reduces the brain specific recurrence rate. [19]. This is in contrast to a report by Mintz *et al.*, which failed to detect a significant beneficial effect of surgical resection [20]. However, the results of this study are controversial, since more than 45% of the patients followed in this trial had uncontrolled systemic disease and 40% presented with a Karnofsky score of 50 or less [9]. In the formerly mentioned trial by Patchell *et al.*, 11% of all patents were found to have non-metastatic lesions, which highlights the importance of surgical resection to confirm the neuropathological diagnosis [10]. In addition to improved survival, surgical resection leads to reduction of mass effects with symptom relief, and decompression of the CSF pathways, especially in the posterior fossa, preventing occlusive hydrocephalus with life threatening complications [21–23]. According to our results, increased ICP and motor impairment such as hemiparesis are specifically amendable to surgical treatment, whereas aphasia and visual deficits are less beneficially influenced. However, since the majority of patients succumb to the exacerbation of their systemic disease, benefit from surgical resection of BM as an invasive strategy associated with significant morbidity, mortality and longer hospital stay was thought to be achieved only if patients have a prognosed life span of more than 6 months [24]. Recent developments such as functional MRI [25], neuro-navigation [26,27] and awake craniotomy [28] have caused a shift of paradigm in clinical neurosciences, including the surgical treatment of BM. The advent of modern technology has revolutionized the pre-operative workup, surgical trajectory planning and intra-operative monitoring with significant benefit to the patients regarding neurofunctional improvement and overall survival. This is reflected by the low morbidity and mortality rates in our study population, which is in accordance to other surgical series [9,29–31]. In addition, we observed significant improvement of the neurological status throughout the entire population independent from RPA classification. This indicates that tailoring the therapeutic decision process solely according to survival–based rating algorithms may not be an adequate strategy [32]. Recent studies have demonstrated that stereotactic radiosurgery (SRS) can lead to excellent tumor control and survival rates comparable to surgical evacuation [29,33,34]. However, since SRS does not primarily reduce mass effects and can induce regressive changes such as intratumoral hemorrhages [35], perifocal edema and radionecrosis [36], this treatment bears specific limitations especially in tumors larger than 3 cm in diameter. Accordingly, a recent study has detected significant treatment–related neurological and non-neurological complications in 40% of 313 patients treated with SRS for BMs [37]. Evidently, SRS is a valid treatment option for patients with small, deep seated or multiple tumors located in surgically inaccessible areas [38]. However, in patients medically suited for surgical intervention, with tumors larger than 2 cm in diameter causing significant mass effects and neurological deficits, surgical evacuation should be considered as a beneficial treatment strategy for each individual patient independent of rigid prognostic indices.

## 5. Conclusions

In addition to short overall survival, BM patients frequently suffer from neurological impairment leading to poor quality of life. Surgical resection causes significant neuro-functional improvement in the majority of BM patients independent from RPA classification. Signs of increased intracranial pressure and motoric impairments are particularly susceptible to microsurgical decompression. Considering the low mortality and morbidity rates, resection should be considered as a valid option to increase neurological function and quality of life for patients with BM.

## Figures and Tables

**Table 1:** Characteristics of patients with brain metastases receiving surgical resection (*n* = 206).

Variable	Number	%
Age (years)		

Mean	61.6	
Range	23.4–83.9	
Gender		

Male	122	59.2
Female	84	40.8
Primary tumor		

Lung cancer	70	34.0
Melanoma	30	14.5
Breast cancer	28	13.6
Colon cancer	20	9.7
Renal cancer	16	7.8
CUP	9	4.4
Urothel cancer	7	3.4
Prostate	4	1.9
Other	22	10.7
Systemic disease		

Controlled	99	48.1
Active	107	51.9
Time of brain metastases		

Synchronous	64	31.1
Metachronous	142	68.9
Status of metastasis		

Solitary	61	29.7
Singular	60	29.1
Multiple	85	41.2

**Table 2:** Medical Research Council-Neurological Performance Status Scale (MRC-NPS).

Grade	Performance
1	No neurological deficit
2	Some neurological deficit but function adequate for useful work
3	Neurological deficit causing moderate functional impairment e.g., ability to move limbs only with difficulty, moderate dysphasia, moderate paresis, some visual disturbance
4	Neurological deficit causing major functional impairment e.g., inability to use limbs, gross speech or visual disturbances
5	No useful function-inability to make conscious responses

**Table 3:** Surgical and neurological morbidity after surgical resection of brain metastases.

Surgical morbidity	Patients	%
CSF leakage	9	4.4
Hemorrhage	6	2.9
Wound infection	3	1.5
Stroke	2	1.0
New seizure	1	0.5
*n*	21	10.3
Neurological morbidity		
New neurological deficit	4	1.9
Worsening of existing deficit	9	4.4
*n*	13	6.3
Total morbidity	34	16.6

**Table 4:** Survival rates stratified by recursive partitioning analysis (RPA) classification.

	Median survival (months)	1-year survival rate (%)	2-year survival rate (%)
all	6.3	24.6	8.2
RPA 1	25.2	43.5	39.1
RPA 2	6.7	22.4	3.7
RPA 3	3.2	21.7	4.3

**Table 5:** Cox regression analysis of prognostic factors for survival.

Parameter	Hazard ratio	95% CI Low	High	*p*
Age	0.02	0.993	1.021	0.882
Tumor size	2.26	0.958	1.282	0.132
Primary tumor	0.06	0.926	1.118	0.801
Metachronous/synchronous	0.56	0.856	1.987	0.454
RPA class	13.70	1.262	2.617	0.001
Solitary/singular/multiple	1.53	0.862	1.308	0.215
Time interval to metastasis	15.50	0.982	1.001	0.001

**Table 6:** Neurological improvement rates at last follow up.

Parameter	Pre-OP *n* (%)	Stable *n* (% affected)	Resolved *n* (% affected)	Improved *n* (% affected)	Worsened *n* (% affected)	*p*
Increased ICP	84 (40.8%)	1 (1.2%)	82 (97.6%)	1 (1.2%)	0	0.001
Hemiparesis	21 (10.2%)	12 (57.1%)	3 (14.3%)	6 (28.6%)	0	0.014
Aphasia	25 (12.1%)	11 (44.0%)	4 (16.0%)	3 (12.0%)	7 (28.0%)	0.334
Visual field defect	21 (10.2%)	18 (85.7%)	1 (4.8%)	0	2 (9.5%)	0.894
